# Rheumatoid arthritis - a neuroendocrine immune disorder: glucocorticoid resistance, relative glucocorticoid deficiency, low-dose glucocorticoid therapy, and insulin resistance

**DOI:** 10.1186/ar4684

**Published:** 2014-11-13

**Authors:** Rainer H Straub

**Affiliations:** 1 Laboratory of Experimental Rheumatology and Neuroendocrine Immunology, Division of Rheumatology, Department of Internal Medicine, University Hospital, 93042 Regensburg, Germany

## 

Neuroendocrine immunology owes much to the foresighted work of Philip S Hench, who together withEdward C Kendall and Tadeus Reichstein introduced glucocorticoids (GCs) into clinical medicine. Since the 1990s, more than 40 years after adding GC to the therapeutic armamentarium, important work has been carriedout to understand GC action. There were three major pathways of discoveries between 1990 and today.

Firstly, the groups of George Chrousos and of Steven Lamberts defined hereditary GC resistance due to abnormalities of the GC receptor (first reviewed in [1,2]). While genetically determined alterations of the GCreceptor were rare in the population, inflammation induced local GC receptor resistance became an important concept of inflammation research [3]. Th e concept gained momentum, particularly in asthma research. Indeed, the local proinflammatory load negatively influences anti-inflammatory GC action [3], but a workable therapeutic approach was not yet introduced into the rheumatology hospital.

Secondly, several groups recognized inadequate secretion of GC in relation to inflammation (reviewed by Maurizio Cutolo and colleagues in this supplement). While an acute inflammatory trigger can increase GCserum levels, this response did not appear after repeated administration of the same trigger [4]. Several studies demonstrated that proinflammatory cytokines such asinterleukin-6, interferon alpha, interferon gamma, and others can stimulate the hypothalamic-pituitary-adrenal axis, but responses to repeated stimuli were much less pronounced. While inadequacy of GC secretion is typical for most active inflammatory diseases, the phenomenon can exist for a long time beyond acute inflammation control (that is, imprinting). In such a situation, long-term therapy with low-dose GC must be recognized as a supplementary therapy for the adrenal glands.

Indeed, between 1990 and today the outstanding role of low-dose GC (approximately 5 mg/day) was discoveredin rheumatoid arthritis (RA) patients using placebo-controlled randomized clinical trials, as summarized in this supplement by Marlies van der Goes and colleagues. In an unstressed individual, the usual daily production of endogenous cortisol mounts to 5.7 mg/m^2 ^(depending on body surface, ≈10 to 14 mg/day) [5], which equals 2.5 to 3.5 mg prednisolone/day. Thus, under real-life conditions, doses of prednisolone between 2 and 5 mg/day represent an adrenocortical substitution therapy. Importantly, low-dose GC has disease-modifying antirheumatic drug effects as demonstrated in this supplement.

Thirdly, timing of daily GC administration reached a new level of precision with a clear pathophysiological concept (reviewed by Cornelia Spies and colleagues). In the early days of GC therapy, doctors and patientsexperienced that GC administration in the evening can sometimes have beneficial effects the following morning. However, these positive effects were accompanied by GC-induced sleep disturbances during the early night. Driven by RA patients' experiences, Arvidson and colleagues overcame this problem by administering GCat 2:00 a.m., and observed a highly beneficial effect in the morning [6]. A more sophisticated method of GC administration was developed during the 2000s - modified GC release, which makes use of GC-loaded pills taken at bedtime and showing abrupt GC release at 2:00 a.m. Under these conditions, GC release occurs at a time when endogenous inflammatory pathways are switched on in a circadian manner [7]. Th e positive effects of nightly GC therapy, as demonstrated in RA[8,9], are due to immediate inhibition of proinflammatory pathways in the increasing flank of nightly inflammation induction [7].

Finally, this supplement presents an article on insulin resistance in chronic inflammatory systemic diseases. While it seems, at first glance, that GCs are not related toinsulin resistance, it turns out that the hypothalamic-pituitary-adrenal axis and thus endogenous GC play an important role in inducing insulin resistance. Pleaserecall that GC can induce elevated levels of glucose, freefatty acids, and glucogenic amino acids in a physiological setting. Insulin resistance is a common phenomenon of physiological states, disease states, and diseases. Th is resistance typically appears in patients with diabetes mellitus, obesity, infection, sepsis, arthritis of different types (including RA), systemic lupus erythematosus, ankylosing spondylitis, trauma, painful states such as postoperative pain and migraine, schizophrenia, major depression, and mental stress. By presenting an integrated theory on insulin resistance, Straub puts forward the concept that insulin resistance is an evolutionarily positively selected program of an activated selfish brain or a stimulated selfish immune system. Long-term use of this adaptive program during chronic activation of the brain or the immune system turns out to be a misguided program that perpetuates the disease process.

## Abbreviations

GC: glucocorticoid; RA: rheumatoid arthritis;

## Competing interests

The author declares that they have competing interests.

## Declaration

This article has been published as part of *Arthritis Research & Therapy* Volume 16 Suppl 2, 2014: At the interface between immunology and endocrinology in rheumatic diseases. The full contents of the supplement are available at http://arthritis-research.com/supplements/16/S2.

This supplement was proposed, developed and commissioned by *Arthritis Research & Therapy *and was funded by an educational grant from Horizon Pharma Inc. All published articles were independently prepared by the authors and have undergone peer review in accordance with the journal's standard policies and processes. Horizon Pharma Inc had no input into the topics covered or the articles themselves. The Supplement Editor was appointed by the journal and declares that they have no competing interests.
